# iTRAQ-Based Quantitative Proteomic Analysis of a Toxigenic Dinoflagellate *Alexandrium catenella* and Its Non-toxigenic Mutant Exposed to a Cell Cycle Inhibitor Colchicine

**DOI:** 10.3389/fmicb.2018.00650

**Published:** 2018-04-04

**Authors:** Shu-Fei Zhang, Yong Zhang, Lin Lin, Da-Zhi Wang

**Affiliations:** ^1^State Key Laboratory of Marine Environmental Science, College of the Environment and Ecology, Xiamen University, Xiamen, China; ^2^Guangdong Provincial Key Laboratory of Fishery Ecology and Environment, South China Sea Fisheries Research Institute, Chinese Academy of Fishery Sciences, Guangzhou, China

**Keywords:** paralytic shellfish toxins, *Alexandrium catenella*, colchicine, cell cycle, toxin biosynthesis, iTRAQ-based proteomics

## Abstract

Paralytic shellfish toxins (PSTs) are a group of potent neurotoxic alkaloids mainly produced by marine dinoflagellates and their biosynthesis is associated with the cell cycle. Study shows that colchicine can cease cell division and inhibit PST production of dinoflagellates. However, the molecular mechanism behind this linkage is unknown. Here, we applied the iTRAQ-based proteomic approach to investigate protein expression profiles of a toxigenic dinoflagellate *Alexandrium catenella* (ACHK-T) and its non-toxigenic mutant (ACHK-NT) when treated with colchicine. The results showed that the cell cycles of both strains were arrested at the G1 phase by colchicine, and the toxin biosynthesis of ACHK-T was inhibited. Among 6,988 proteins identified, 113 and 253 proteins were differentially expressed in the colchicine-treated ACHK-T and ACHK-NT, respectively, compared with their non-colchicine treatments. Proteins involved in reactive oxygen species scavenging and protein degradation were upregulated in both strains while proteins participating in photosynthetic pigment biosynthesis and nitrogen metabolism presented different expressions. Nitrate reductase and glutamine synthetase were altered insignificantly in the colchicine-treated ACHK-T while both of them were remarkably downregulated in the colchicine-treated ACHK-NT, suggesting a feedback regulation between PST production and nitrogen metabolism in ACHK-T. Nitrogen originally for PST biosynthesis might be reallocated to photosynthetic pigment biosynthesis in the colchicine-treated ACHK-T. A total of 55 homologs of 7 toxin-related proteins were obtained; however, they altered insignificantly in both colchicine-treated strains, suggesting that toxin biosynthesis might be post-translationally regulated. Our study provided new insights into toxin biosynthesis in marine dinoflagellates.

## Introduction

Paralytic shellfish toxins (PSTs) are a group of neurotoxic alkaloids which selectively block the voltage-gated sodium channels in excitable cells and result in paralytic shellfish poisonings around the world. It is estimated that PSTs result in about 2,000 poisoning incidents with 15% mortality annually, and they have become a global public health concern (Hallegraeff, 1993; Anderson et al., 2012).

The PSTs are produced by two different kingdoms of life: prokaryotic cyanobacteria and eukaryotic dinoflagellates (Cusick and Sayler, 2013). In toxigenic cells, PST is an important nitrogen-rich macromolecule besides proteins and nucleic acids. The parental substrate of PST, saxitoxin is composed of 33% nitrogen on a molecular weight basis and toxin production is thought to be a result of a specific pathway integrated in the general nitrogen metabolism of the toxigenic cells (Taroncher-Oldenburg et al., 1999). The biosynthetic pathway of PSTs has been elucidated in several cyanobacterial species such as *Cylindrospermopsis raciborskii*, *Anabaena circinalis*, *Aphanizomenon* sp., and *Lyngbya wollei* (Kellmann et al., 2008; Mihali et al., 2009; Stucken et al., 2010; Mihali et al., 2011). A group of core genes (*sxtA*, *sxtG*, *sxtB*, *sxtD*, *sxtS*, *sxtU*, *sxtH/T*, and *sxtI*) directly involved in toxin biosynthesis, tailor genes (*sxtI*, *sxtN*, *sxtX*) participating in toxin transformation, as well as some additional genes responsible for toxin transportation and regulation, have been characterized in cyanobacteria, and several toxin-related proteins have also been identified (Kellmann et al., 2008; D’Agostino et al., 2014). In contrast, toxin biosynthesis in dinoflagellates remains obscure although the pathway is believed to be similar to that in cyanobacteria (Shimizu, 1993). Some putative homologs of cyanobacterial toxin genes and proteins have been identified in dinoflagellates (Hackett et al., 2013; Zhang et al., 2015), however, only *sxtA* and *sxtG* participating in the first two biosynthetic steps are well characterized (Stüken et al., 2011; Orr et al., 2013).

Previous studies indicate that toxin production is coupled to the cell cycle (Taroncher-Oldenburg et al., 1997, 1999). In toxigenic dinoflagellates, PST production is not successive but a discontinuous biological process coupled to a restrained time frame of the cell cycle (Taroncher-Oldenburg et al., 1997). Three genes related to biosynthesis of PST precursors are upregulated during the toxin producing phase (Harlow et al., 2007), and the expressions of nine proteins potentially involved in toxin biosynthesis vary significantly at different toxin biosynthesis phases in *Alexandrium catenella* within a cell cycle (Wang et al., 2013a). Recent studies indicate that some metabolic inhibitors can affect cell cycle and PST biosynthesis. In *Alexandrium tamarense*, colchicine prolongs the G1 phase and inhibits toxin production which originally occurs in the S phase (Cho et al., 2011). Both mitomycin C and 5-fluoro-2′-deoxyuridine can arrest *A. tamarense* at the S phase but toxin production continues (Cho et al., 2011, 2014). However, the molecular mechanism behind this linkage to cell cycle and toxin production is not clear.

In our previous study, a non-toxic mutant of the toxigenic *A. catenella* (ACHK-NT) is obtained from a parental toxic strain (ACHK-T) isolated from the South China Sea (Wang and Hsieh, 2005). Comparison of transcriptoms and proteomes between these two strains and in different toxin biosynthesis stages reveals genes and proteins potentially involved in PST biosynthesis, and different carbon and energy utilization strategies result in the different ability of toxin production (Zhang et al., 2014, 2015, 2017). Moreover, toxin biosynthesis of ACHK-T is correlated to cell cycle and occurs just in a restricted period of G1 phase. Here, we investigated the protein expression profiles of ACHK-T and ACHK-NT exposed to colchicine using the iTRAQ-based quantitative proteomic approach. Our goal was to unveil response mechanisms of *A. catenella* to colchicine and to mine molecular processes associated with PST biosynthesis at the protein level.

## Materials and Methods

### Organisms and Culture Conditions

Unialgal cultures of toxic (ACHK-T) and non-toxic (ACHK-NT) *A. catenella* strains were provided by the Collection Center of Marine Algae, Xiamen University. Cultures of ACHK-T and ACHK-NT were maintained in *K*-medium (Keller et al., 1987) at 20°C. An irradiance of approximately 100 μE⋅m^-2^⋅s^-1^ was provided using cool white fluorescent bulbs under a 14:10-h light:dark photoperiod.

Synchronization of ACHK-T and ACHK-NT cells was conducted using the method as reported (Shi et al., 2013). Briefly, the active cells floating in the upper layer were inoculated into new fresh medium with cell suspension:medium = 1:3 every 3 days. After repeating this for a month, cells of the two strains were finally transferred into 12 5-L flasks filled with 4-L *K*-medium, 6 flasks for ACHK-T and 6 for ACHK-NT. For each strain, colchicine was added to three flasks with a final concentration of 2.5 mM, while the other three flasks were left untreated as a control. Sample collections were started at 08:00 of the next day of inoculation and carried out every 2 h for 24 h except for proteomic analysis. At each sampling time point, 1 mL portions of each culture were collected in 1.5 mL tubes with Lugol’s solution for cell counts; 50 mL of the culture from each flask was collected by centrifugation, suspended in 1 mL 70% ethanol and stored at -20°C for cell cycle analysis; and 50 mL of each ACHK-T culture was collected by centrifugation and stored at -20°C for toxin analysis. Cells at 02:00 were harvested for proteomic analysis, at which toxin content increased sharply according to our toxin analysis result. A total of 500 mL of each culture (approximate 10^6^ cells) was collected by centrifugation, resuspended in 1 mL TRIzol Reagent, frozen in liquid nitrogen and stored at -80°C.

### Toxin Analysis

The PST analysis was conducted following a previously reported protocol (Wang et al., 2012). Briefly, cells were homogenized with sonication in 0.5 mL of 50 mM acetic acid. The supernatant obtained after centrifugation at 10,000 g for 30 min was filtered with 0.22 μm pore filter membranes (Millipore). A total of 10 μL filtrate was subjected to HPLC-fluorescence analysis with an Intersil C8-5 column. The concentration of PST in each sample was determined through comparing the peak areas of sample with the toxin standards for gonyautoxin (GTX) and C1/C2, which were bought from the National Research Council, Canada.

### Flow Cytometric Analysis

Flow cytometric analysis was conducted following a previously reported protocol (Wang et al., 2013a). Briefly, cells were centrifuged at 10,000 g for 5 min, washed twice with 1 × phosphate buffer saline (PBS, 137 mM NaCl, 2.7 mM KCl, 10 mM Na_2_HPO_4_, 2 mM KH_2_PO_4_, pH = 8.0), incubated in 100 μL RNase A for 10 min and then stained with 2.5 mg/mL propidium iodide in 1 mL 1 × PBS. The DNA content of each sample was analyzed with an Epics XL flow cytometer (Bechman Coulter, United States) and the cell cycle was determined based on the histograms of relative DNA content.

### Protein Preparation

Proteins were extracted following a previously reported protocol (Wang et al., 2012). Briefly, cells were homogenized with sonication and protein was extracted using TRIzol reagent (Invitrogen, Carlsbad, CA, United States), chloroform and ethanol, precipitated using isopropanol and washed with 95% ethanol and air-dried. A total of 100 μL of rehydration buffer was added to dissolve the air-dried pellet. Then the proteins were reduced with 10 mM DTT at 56°C for 1 h, alkylated by 55 mM iodacetamide, and precipitated by 4 × volume of acetone. After air drying, the pellet was dissolved in 500 μL 0.5 M tetraethyl-ammonium bromide (TEAB) (Applied Biosystems, Milan, Italy) and quantified with Bradford assay using BSA as standard.

### Peptide Labeling

For 8-plex iTRAQ labeling, control and colchicine-treated groups of ACHK-T and ACHK-NT were compared using two biological replicates for each. A total of 100 μg protein from each sample was digested using Trypsin Gold (Promega, Madison, WI, United States) with the ratio of protein:trypsin = 30:1 at 37°C for 16 h. The trypsin-digested samples were dried by vacuum centrifugation and then reconstituted in 0.5 M TEAB. Each tryptic digest was labeled in accordance with the manufacturer’s instructions with one isobaric amine-reactive tag as follows: ACHK-T1 (113 tag), ACHK-NT1 (114 tag), colchicine-treated ACHK-T1 (115 tag), colchicine-treated ACHK-NT1 (116 tag), ACHK-T2 (117 tag), ACHK-NT2 (118 tag), colchicine-treated ACHK-T2 (119 tag), and colchicine-treated ACHK-NT2 (121 tag). After 2 h incubation, the labeled samples were pooled and dried using vacuum centrifugation.

### Cation Exchange Fractionation

The dried samples were reconstituted with 4 mL buffer A (25 mM NaH_2_PO_4_ in 25% ACN, pH 2.7) and were used for cation exchange fractionation using an LC-20AB HPLC pump system (Shimadzu, Kyoto, Japan). The peptides were eluted at a flow rate of 1 mL/min with a gradient of buffer A for 10 min, 5–60% buffer B (25 mM NaH_2_PO_4_, 1 M KCl in 25% ACN, pH 2.7) for 27 min, and 60–100% buffer B for 1 min. The system was then maintained at 100% buffer B for 1 min before equilibrating with buffer A for 10 min prior to the next injection. Elution was monitored by measuring the absorbance at 214 nm, and fractions were collected every 1 min. The eluted peptides were pooled into 20 fractions, desalted with a Strata X C18 column (Phenomenex) and vacuum-dried, and then reconstituted in buffer C (5% ACN, 0.1% formic acid).

### LC-MS/MS Analysis

All peptide samples were separated on an LC-20AD nanoHPLC (Shimadzu, Kyoto, Japan) system and analyzed on an AB SCIEX TripleTOF 5600 System. The samples were loaded at 8 μL/min for 4 min, then a 35 min gradient was run at 300 nL/min starting from 2 to 35% buffer D (95% ACN, 0.1% formic acid), followed by a 5 min linear gradient to 60%, then, a 2 min linear gradient to 80%, maintained at 80% for 4 min, and finally returned to 5% within 1 min.

The MS was operated with a RP of ≥ 30,000 full width at half maximum (FWHM) for TOF MS scans. For IDA, survey scans were acquired in 250 ms and as many as 30 product ion scans were collected if exceeding a threshold of 120 counts per second and with a 2+ to 5+ charge-state. Total cycle time was fixed to 3.3 s. The Q2 transmission window was 100 Da for 100%. Four time bins were summed for each scan at a pulse frequency value of 11 kHz through monitoring of the 40 GHz multichannel TDC detector with four-anode/channel detection. A sweeping collision energy setting of 35 ± 5 eV coupled with iTRAQ adjust rolling collision energy was applied to all precursor ions for collision-induced dissociation. Dynamic exclusion was set for half of the peak width (15 s), and then the precursor was refreshed off the exclusion list.

### Bioinformatics Analysis

The database used for protein identification contained amino acid sequences translated from unigenes in three projects: the transcriptomes of ACHK-T and ACHK-NT (Zhang et al., 2014), the transcriptomes of ACHK-T from different cell cycle phases (Zhang et al., 2017), and the transcriptomes of samples as in this study (unpublished data). MASCOT genetic format files converted from raw data files using Proteome Discoverer 1.2 (PD 1.2, Thermo) were searched and protein identification was performed using the MASCOT search engine (Matrix Science; version 2.3.02). The search parameters were as follows: fragment mass tolerance at 0.1 Da; peptide mass tolerance at 0.05 Da; trypsin as the enzyme, up to one missed cleavage allowed; and peptide charges of 2+ and 3+. The iTRAQ labeling and carbamidomethylation were defined as fixed modifications. The Gln- > pyro-Glu (N-term Q), Oxidation (M), and Deamidated (NQ) were the potential variable modifications. Specifically, an automatic decoy database search was performed to estimate the false discovery rate (FDR). Peptides at the 95% confidence interval were counted as identified, and each confident protein identification involved at least one unique peptide.

Functional annotations of proteins were conducted using the Blast2GO program against the Kyoto Encyclopedia of Genes and Genomes (KEGG) and the National Center Biotechnology Information non-redundant protein sequences database (NCBInr) with a criterion of *e*-value ≤ 1E-5.

The quantitative protein ratios were weighted and normalized by the median ratio in Mascot. Proteins were compared between two pairs: ACHK-T vs. colchicine-treated ACHK-T and ACHK-NT vs. colchicine-treated ACHK-NT. A protein was considered to be differentially expressed if it contained at least two unique peptides, had similar and significant ratios of ≥ 1.2 (upregulated) or ≤ 0.83 (downregulated) in both of the replicates (D’Agostino et al., 2014; Li et al., 2016; Wang et al., 2016).

### Identification of Toxin-Related Proteins

To identify toxin-related proteins, BLAST was performed with toxin-related protein sequences of the cyanobacterium *C. raciborskii* T3 (Kellmann et al., 2008) against the proteins identified. All hits with an *e*-value ≤ 1E-5 were retrieved as confident identifications.

## Results

### Effects of Colchicine on Cell Cycle and Toxin Content

**FIGURE 1:**
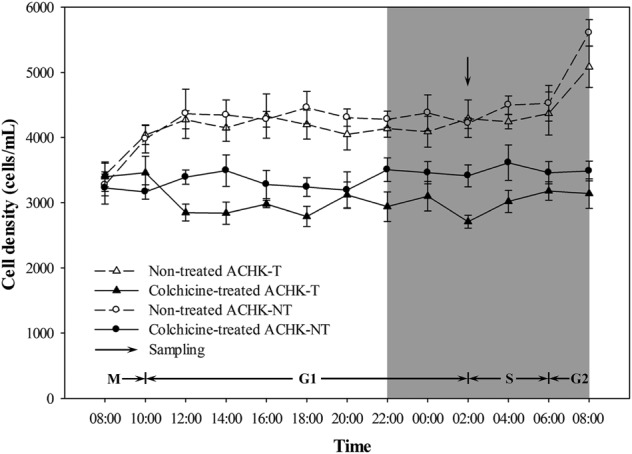
Variation of cell density during a cell cycle of ACHK-T and ACHK-NT with and without colchicine. The cell counts were reported as the mean of biological triplicates with standard deviation.

**FIGURE 2:**
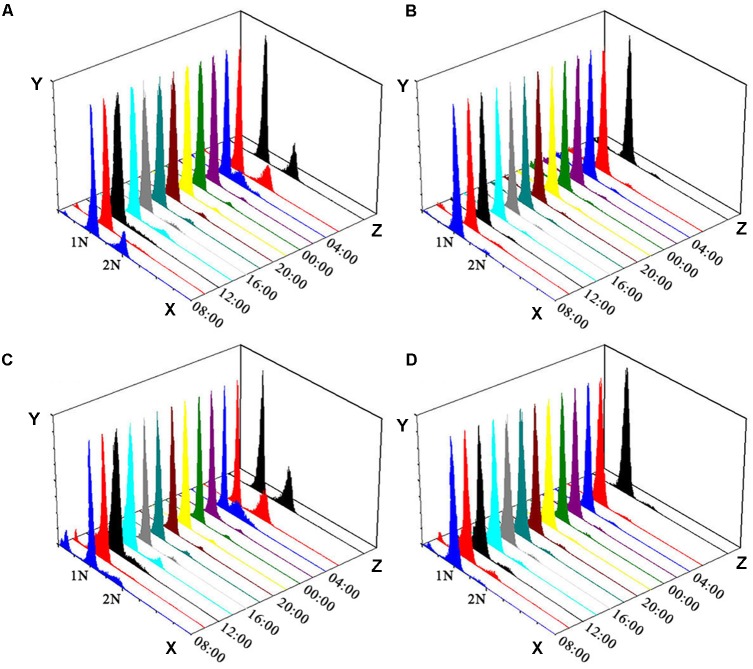
Flow cytometry analysis of ACHK-T and ACHK-NT with and without colchicine. **(A)** Non-treated ACHK-T (control); **(B)** Colchicine-treated ACHK-T; **(C)** Non-treated ACHK-NT (control); **(D)** Colchicine-treated ACHK-NT. *X*-axis: Relative DNA content; *Y*-axis: Cell number; *Z*-axis: Time.

Both colchicine-treated ACHK-T and ACHK-NT cultures ceased cell division and cell densities maintained relatively stable within one diel cycle, while cell division of non-treated cultures occurred from 06:00 to 10:00 (G2/M phase) and cell densities increased significantly (**Figure 1**). The representative DNA histograms of the non-treated and colchicine-treated ACHK-T and ACHK-NT within one diel cycle are shown in **Figure 2**. Non-treated cells of both strains presented discrete G1, S, and G2/M phases during a light-dark cycle (**Figures 2A,C**). The G1 phase started from 10:00 and lasted until 02:00 of the next day, cells then entered the S phase followed by G2/M phase (mitosis) which lasted approximately 4 h (06:00–10:00). However, cells of both strains were arrested at the G1 phase by colchicine; and the DNA histograms of these colchicine-treated cultures were identical within a diel cycle.

**FIGURE 3:**
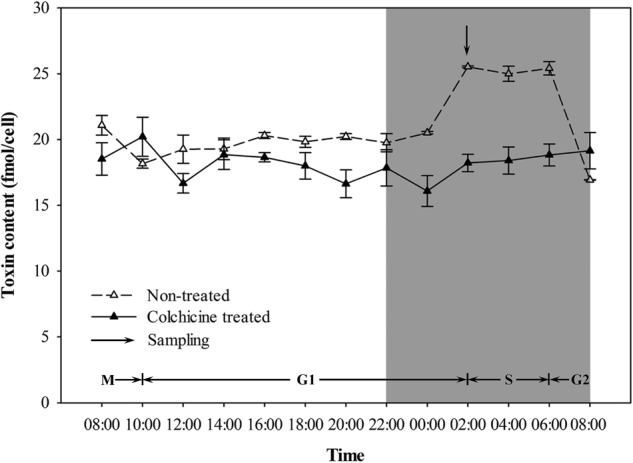
Variation of toxin content during a cell cycle of ACHK-T with and without colchicine. The toxin contents were reported as the mean of biological triplicates with standard deviation.

Variations in the toxin contents of the non-treated and colchicine-treated ACHK-T within a diel cycle are shown in **Figure 3**. Toxin content of the non-treated ACHK-T presented a periodical variation: it increased rapidly from 00:00 to 02:00 (G1 phase) and decreased sharply when cells divided from 06:00 to 08:00 (G2/M phase). In contrast, toxin content of the colchicine-treated ACHK-T varied only a little and maintained a relatively stable level during the whole cell cycle (**Figure 3**).

### Proteome Overview and Protein Annotation

Overall, 50,903 of the whole 457,096 output spectra were matched to 19,469 peptides with an approximately 11.1% spectrum utilizing rate. Using the Mascot search engine, 6,988 proteins were identified from 16,830 unique peptides that collectively matched 37,862 unique spectra. All proteins identified were annotated using the KEGG and NCBInr databases (Supplementary Table 1).

**FIGURE 4:**
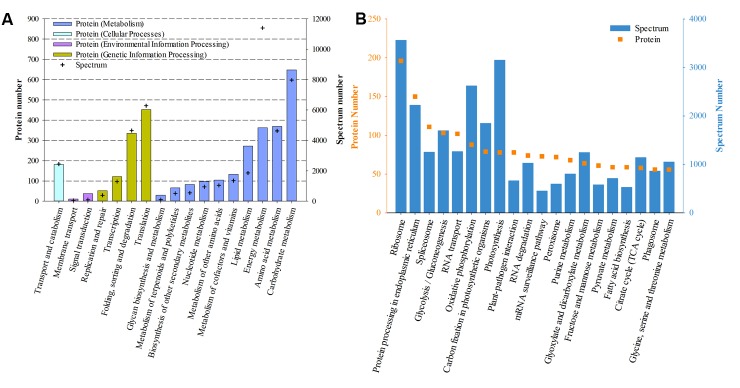
Functional distribution of proteins based on KEGG annotation. **(A)** Based on the secondary hierarchy; **(B)** Top 20 abundant (according to protein number) pathways based on the third hierarchy.

Based on the KEGG categories, proteins were annotated into 17 groups. “Carbohydrate metabolism,” “Amino acid metabolism,” and “Energy metabolism” ranked as the top three abundant categories in “Metabolism,” while “Translation,” and “Folding sorting and degradation” dominated the “Genetic Information Processing” (**Figure 4A**). Further classification of the third pathway hierarchy showed that “Ribosome,” “Protein processing in endoplasmic reticulum,” and “Spliceosome” were the most frequently detected pathways (**Figure 4B**).

To further annotate protein function, all proteins were subjected to the NCBInr database. A total of 4,543 proteins were successfully assigned with diverse functional annotations (Supplementary Table 1). Proteins involved in photosynthesis (based on spectrum number) were the most abundant, such as peridinin-chlorophyll a-protein, chlorophyll a/c–binding protein and ribulose 1,5-bisphosphate carboxylase oxygenase. In addition, histone-like protein, ATP synthase and bioluminescence-related proteins were also presented in the list of the top abundant proteins.

### Differentially Expressed Proteins

**FIGURE 5:**
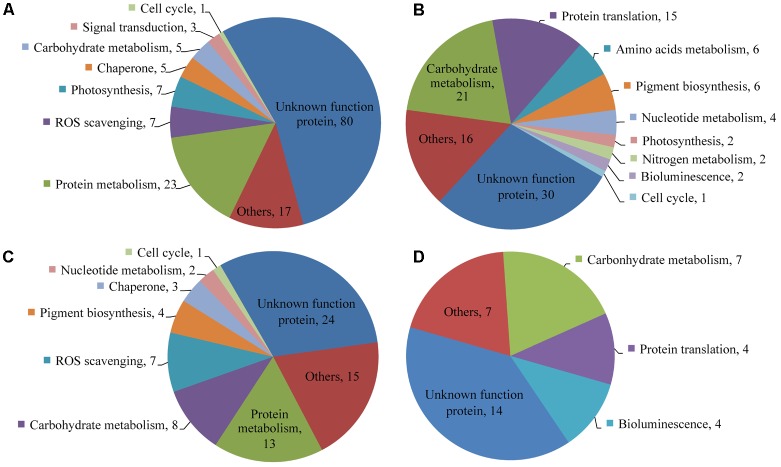
Functional distribution of differentially expressed proteins. **(A)** Upregulated proteins in the colchicine-treated ACHK-NT; **(B)** Downregulated proteins in the colchicine-treated ACHK-NT; **(C)** Upregulated proteins in the colchicine-treated ACHK-T; **(D)** Downregulated proteins in the colchicine-treated ACHK-T.

Of the 6,988 proteins identified, 113 proteins in the colchicine-treated ACHK-T and 253 in the colchicine-treated ACHK-NT presented differential expressions. In the former, 77 proteins were upregulated and 36 proteins were downregulated; while in the latter, 148 proteins were upregulated and 105 proteins were downregulated. Functional classifications of these proteins based on KEGG and NCBInr annotation are presented in **Figure 5** and Supplementary Table 2.

In the colchicine-treated ACHK-T and ACHK-NT, the functional distributions of the upregulated proteins were similar. A large number of them were involved in protein degradation and the protein folding process, including 20S proteasome subunits, Lon-like ATP-dependent protease, cathepsin, legumain, and phytepsin, and the reactive oxygen species (ROS) scavenging process, e.g., glutathione peroxidase (GPx), Cu/Zn superoxide dismutase (SOD1), catalase-peroxidase (katG), peptide-methionine (R)-S-oxide reductase, glutathione S-transferase (GST), L-ascorbate peroxidase (APX), and cytochrome c peroxidase (CCP). For the downregulated proteins, most of them were assigned to carbohydrate metabolism and protein translation, such as translation initiation factor 3, elongation factors Ts and 1-gamma, and ribosomal proteins. The bioluminescence-related proteins (luciferase and luciferin binding protein, LBP) were also downregulated in both colchicine-treated strains.

Four proteins involved in chlorophyll and carotenoid biosynthesis were upregulated in the colchicine-treated ACHK-T while six proteins participating in these processes were downregulated in the colchicine-treated ACHK-NT. Moreover, two key enzymes involved in nitrogen metabolism, nitrate reductase (NR) and glutamine synthetase (GS), were downregulated in the colchicine-treated ACHK-NT, while their expressions altered insignificantly in the colchicine-treated ACHK-T. It should be noted that a large number of differentially expressed proteins were annotated with unknown functions, and these proteins might have been involved in toxin biosynthesis or other biological processes.

### Toxin-Related Proteins

With a cutoff *e*-value ≤ 1E-5, a total of 55 homologs of seven toxin-related proteins were obtained: sxtG, sxtH, sxtO, sxtT, sxtU, sxtZ, and ompR. However, all of them altered insignificantly in both colchicine-treated strains (**Table 1**).

**Table 1:** Identification and quantitation of toxin-related proteins in *Alexandrium catenella*.

Protein	Homologs	Top hit protein	Top score	*e*-value	Putative function	T/C (ACHK-NT)		T/C (ACHK-T)	
							
						Ratio 1	Ratio 2	Ratio 1	Ratio 2
sxtG	1	CL1611.Contig1_All	76	5E-18	Amidinotransferase	0.71^∗^	1.11	1.26	1.03
sxtH	3	comp55174_c0_orf1	84	2E-18	Phenylpropionate dioxygenase	–	–	–	–
sxtO	2	comp43217_c0_orf1	181	4E-58	Adenylylsulfate kinase	0.94	0.93	0.98	0.92
sxtT	2	comp55174_c0_orf1	85	1E-19	Phenylpropionate dioxygenase	–	–	–	–
sxtU	45	Unigene66541_All	134	6E-39	Short-chain alcohol dehydrogenase	1.95	1.68	1.24	1.54
sxtZ	1	comp61883_c0_orf1	55	9E-9	Signal transduction	–	–	–	–
ompR	1	comp17794_c0_orf1	72	1E-14	Signal transduction	–	–	–	–


*T/C: colchicine-treated/control group; –: unquantifiable; ^∗^*p*-value ≤ 0.05.*

## Discussion

In this study, we compared protein expression profiles of the colchicine-treated and non-treated ACHK-T and ACHK-NT strains using the iTRAQ-based proteomic approach. A total of 113 and 253 proteins presented differential expressions in the colchicine-treated ACHK-T and ACHK-NT strains, respectively. These proteins were involved in various biological processes, i.e., cell cycle regulation, reactive oxygen species scavenging, protein metabolism, photosynthetic pigment biosynthesis, and nitrogen metabolism, but some of them presented different variations between the two strains, which might be caused by their different toxin producing abilities. The ACHK-T cells had the ability to produce toxin but toxin synthesis was inhibited by colchicine, suggesting that colchicine affected toxin synthesis–related proteins or biological processes; however, the ACHK-NT cells lost the ability to synthesize toxin and toxin synthesis pathway no longer existed in the cells, thus colchicine affected other biological processes.

### Cell Cycle

It is reported that a high concentration of colchicine is able to prolong the G1 phase of *A. tamarense*, even though colchicine arrests several other eukaryotic cell types in the M phase (Cho et al., 2011). In our study, we found that the cells of both ACHK-T and ACHK-NT were arrested at the G1 phase when treated with colchicine, which was similar to the results recorded for *A. tamarense* (Cho et al., 2011).

Two proteins participating in cell cycle regulation, S phase kinase-associated protein 1 (SKP1) and proliferating cell nuclear antigen (PCNA), were identified. The former was upregulated in both colchicine-treated strains while the latter was significantly downregulated only in the colchicine treated ACHK-NT. In eukaryotic cells, SKP1 is an important protein required for the ubiquitin-mediated proteolysis of some cell-cycle regulatory proteins and is indispensable for the G1/S and G2/M transitions (Bai et al., 1996; Yu et al., 1998). Moreover, SKP1 is also involved in transcriptional regulation, signal transduction, and many other cellular processes in eukaryotes. PCNA is a factor of DNA polymerase, participating in DNA synthesis, maturation, and repair. Moreover, it also plays an essential role in cell cycle regulation and is required for G1 progression and G1/S transition (Strzalka and Ziemienowicz, 2011). Both the PCNA gene and the protein are studied in several dinoflagellate species and their expressions fluctuate regularly during a cell cycle (Zhao et al., 2009; Brunelle and Van Dolah, 2011; Wang et al., 2013b). Therefore, differential expressions of these two proteins should partially account for cell cycle arrest at the G1 phase in the two *A. catenella* strains.

### Toxin Production

In our study, the toxin content of the colchicine-treated ACHK-T maintained a low, stable level compared to the non-treated ACHK-T, which presented a sharp increase in the late G1 phase (**Figure 3**). This result was similar to the result of the colchicine-treated *A. tamarense* whose toxin production occurs in the S phase (Cho et al., 2011). According to a previous study, toxin production of *A. tamarense* is inhibited when colchicine prolongs the non-toxin biosynthetic phase (G1 phase), while toxin content increases gradually when mitomycin C arrests the cells in the toxin biosynthetic phase (S phase) (Cho et al., 2011). Moreover, it is reported that the G1 phase of *Alexandrium* can be divided into two periods: early G1 and late G1 phases (Taroncher-Oldenburg and Anderson, 2000). Therefore, it could be postulated that the cell cycle of ACHK-T might be arrested in the non-toxin biosynthetic G1 phase (**Figure 3**) if the inhibition of toxin production was caused by cell cycle arrest. However, we could not rule out the possibility that colchicine might inhibit toxin biosynthesis directly (Cho et al., 2014) or affect some biological processes related to toxin biosynthesis other than cell cycle.

With BLAST searching, we screened out 55 confidential homologs of seven toxin-related proteins among the proteins identified, which was similar to our previous study (Zhang et al., 2014). SxtG, sxtH/T, and sxtU are directly involved in PST biosynthesis; sxtO is related to the conversion of PST analogs; and sxtZ and ompR are related to transcriptional regulation of PST synthesis (Kellmann et al., 2008). However, quantitative analysis showed no significant abundance variations of these toxin-related proteins in both colchicine-treated strains, although the toxin contents were significantly different between the colchicine-treated and non-treated ACHK-T. It could be postulated that toxin biosynthesis might be regulated at the post translational level, which was in agreement with the previous speculation that toxin biosynthesis in *A. catenella* might be regulated at the translational or post-translational level (Kellmann and Neilan, 2007).

### ROS Scavenging

Cells can generate ROS as cellular signaling molecules, and the production and scavenging of ROS maintains an equilibrium state (D’Autreaux and Toledano, 2007). However, this equilibrium can be perturbed by a number of adverse abiotic stress factors, resulting in excessive ROS which damages lipids, proteins, and nucleic acids (Apel and Hirt, 2004). In our study, a large number of proteins involved in ROS scavenging were upregulated in both colchicine-treated strains (**Figure 5** and Supplementary Table 2). Most of them (GPx, katG, APX, CCP, and SOD1) catalyze H_2_O_2_ and O_2_^-^ into H_2_O and O_2_ (Apel and Hirt, 2004). GST catalyzes mainly the conjugation of the reduced form of glutathione to xenobiotic substrates for the purpose of detoxification and it plays an important role in herbicide detoxification in plants (Marrs, 1996; Dixon et al., 2002). In addition, another protein (mitochondrial alternative oxidase 1c), which limits the production of ROS (Hanqing et al., 2010), was upregulated in both colchicine-treated strains. These results were similar to those in the proteomic study of the colchicine-treated *Ginkgo biloba* L, in which three proteins involved in ROS scavenging and detoxifying are upregulated after colchicine treatment (Yang et al., 2013). Overall, the higher expressions of these proteins suggested that colchicine treatment elevated the intracellular ROS level, and the cells detoxified ROS toxicity via enhancing the expression of these proteins.

### Bioluminescence

Dinoflagellates are the main eukaryotic protists capable of producing light in the marine environment (Widder, 2010). The production of light in dinoflagellates occurs in scintillons which contain luciferin substrate, luciferase enzyme (LCF), and LBP (Nicolas et al., 1987). In our study, the bioluminescence participants LCF and LBP were significantly downregulated in both colchicine-treated ACHK-T and ACHK-NT, indicating that colchicine inhibited the biosynthesis of LCF and LBP, which might affect the bioluminescence of *A. catenella*. It is reported that both LCF and LBP are circadian-regulated at the translation level in marine dinoflagellates (Johnson et al., 1984; Morse et al., 1989). The arrest of cell cycle at G1 phase by colchicine might disturb the normal circadian rhythm of *A. catenella*, which subsequently affected the biosynthesis of LCF and LBP. This postulation needs further study.

### Protein Metabolism

Among the differentially expressed proteins, a large number of proteases involved in protein degradation were upregulated in both colchicine-treated ACHK-NT and ACHK-T (**Figure 5** and Supplementary Table 2). In plants, these proteins participate in protein maturation, degradation, and protein rebuilding in response to various external stimuli and also play a housekeeping function to remove abnormal misfolded proteins (Beers et al., 2000; Kidrič, 2014). Our result was similar to those in the proteomic study of the colchicine-treated *Ginkgo biloba* L (Yang et al., 2013). In addition, disulfide-isomerase A6 involved in protein folding and several molecular chaperones were upregulated in both colchicine-treated strains. Two proteins, peptidylprolyl isomerase and cyclophilin B, and two chaperone GrpE and FK506-binding protein 2 were upregulated in the colchicine-treated ACHK-NT. These proteins and chaperones regulate various biological processes, especially protein folding (Fink, 1999). Therefore, upregulation of these proteins indicated that colchicine caused an abiotic stress on these two strains, resulting in the enhancement of protein misfolding and degradation.

On the other hand, several proteins involved in protein translation were downregulated in both colchicine-treated strains. These proteins participate in translation process or involve in the biosynthesis of substrates for protein translation (Kapp and Lorsch, 2004). In addition, replication factor A1 (Longhese et al., 1994) was downregulated in the colchicine-treated ACHK-NT and RNA-binding protein 8A in ACHK-T. They participate mainly in the synthesis and maturation of nucleic acids involved in protein translation. Downregulation of these proteins suggested that colchicine might suppress protein translation in these two strains.

### Photosynthetic Pigment Biosynthesis

In dinoflagellates, chlorophyll and carotenoid are the two major groups of pigments although their composition pattern varies among different species (Jeffrey et al., 1975). The biosynthesis process of chlorophyll can be divided into three parts: formation of 5-aminolevulinic acid, formation of protoporphyrin IX, and formation of chlorophyll in the magnesium branch (Eckhardt et al., 2004). In our study, among the differentially expressed proteins of the colchicine-treated ACHK-NT, four proteins are involved in chlorophyll biosynthesis: hydroxymethylbilane synthase (hemC) participating in the second part, magnesium chelatase subunit I and geranylgeranyl reductase in the third part, and chlorophyll (ide) b reductase (NOL) in the biotransformation from chlorophyll b to chlorophyll a (Porra, 1997; Eckhardt et al., 2004). Interestingly, all of them were downregulated in the colchicine-treated ACHK-NT, indicating that chlorophyll biosynthesis was inhibited by colchicine. However, among the differentially expressed proteins of the colchicine-treated ACHK-T, hemC and NOL are the only two proteins involved in chlorophyll biosynthesis and both of them were upregulated. Moreover, two proteins: xanthoxin dehydrogenase and (+)-neomenthol dehydrogenase involved in the biosynthesis of carotenoid and its substrates were upregulated in the colchicine-treated ACHK-T, while another two proteins with similar functions were downregulated in the colchicine-treated ACHK-NT (Cunningham et al., 1998). These results indicated that the response of the two strains to colchicine was discrepant and biosynthesis of photosynthetic pigment was enhanced in the colchicine-treated ACHK-T.

### Nitrogen Metabolism

During nitrogen metabolism, ammonia (NH_4_^+^) is incorporated as an amide group of glutamine in the GS/glutamate synthase pathway, while nitrate (NO_3_^-^) should be reduced to nitrite (NO_2_^-^) via NR and NO_2_^-^ is then reduced to NH_4_^+^ by nitrite reductase (Wheeler, 1983). In our study, two key enzymes involved in nitrogen metabolism (NR and GS) were downregulated in the colchicine-treated ACHK-NT. However, among the differentially expressed proteins of the colchicine-treated ACHK-T, no enzymes directly involved in nitrogen metabolism were found. These results indicated that the nitrogen metabolism of the two strains was different in response to colchicine treatment. Considering the large percentage of nitrogen in the PST molecule and the relationship between toxin production and nitrogen metabolism (Taroncher-Oldenburg et al., 1999), the different intracellular toxin contents of these two strains should be one key factor responsible for this discrepancy.

**FIGURE 6:**
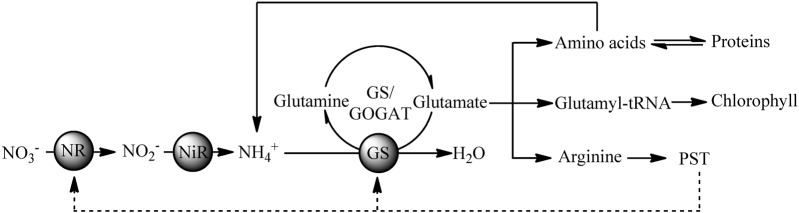
Putative relationships between PST production and nitrogen metabolism in the toxigenic dinoflagellate *Alexandrium catenella*.

Previous studies reveal that toxin production is correlated to cell cycle and intracellular toxin content fluctuates within a certain range (Taroncher-Oldenburg et al., 1997; Taroncher-Oldenburg and Anderson, 2000; Wang et al., 2013a). This suggests that there might be a feedback regulation mechanism between intracellular toxin content and toxin production, and NR and GS might be the regulative points. In our study, the toxin content of the colchicine-treated ACHK-T was maintained at a low level within the whole cell cycle, and the toxin content collected at time point 02:00 was significantly lower than that of non-treated ACHK-T (**Figure 3**). Since toxin production occurred mainly in the period 00:00–02:00, the signal of low toxin content might produce a negative feedback on the nitrogen metabolism process (**Figure 6**) which kept the expressions of NR and GS away from the downregulation which occurred in the colchicine-treated ACHK-NT. In order to balance the intracellular nitrogen, the colchicine-treated ACHK-T cells might allocate nitrogen originally for PST biosynthesis to synthesize photosynthetic pigments, since biosynthesis of nitrogenous chlorophyll and carotenoid are closely subjected to nitrogen assimilation (Yentsch and Vaccaro, 1958; Mishra and Srivastava, 1983; Sinha et al., 1988) and this process was abnormally enhanced in the colchicine-treated ACHK-T.

## Conclusion

Our study, for the first time, investigated the effects of colchicine on cell cycle and toxin production of a toxigenic *A. catenella* and its non-toxic mutant strain, and compared global protein expression profiles using the iTRAQ-based quantitative proteomic approach. Colchicine arrested cells of both strains at the G1 phase and inhibited toxin production of ACHK-T. ROS scavenging and protein degradation were enhanced in both colchicine-treated strains while photosynthetic pigment biosynthesis and nitrogen metabolism presented different responses between the two strains. Inhibition of toxin production might produce a feedback regulation on nitrogen metabolism, and nitrogen originally intended for PST biosynthesis was utilized to synthesize photosynthetic pigments in the colchicine-treated ACHK-T. Although seven toxin-related proteins were identified, they altered insignificantly in both colchicine-treated strains, suggesting the post-translational regulation of toxin biosynthesis. Overall, our study provided new insights into the PST biosynthesis of dinoflagellates and shed light on the tangled relationships between cell cycle and toxin production.

## Author Contributions

D-ZW and S-FZ planned and designed the research. S-FZ, YZ, and LL performed the experiments and analyzed the data. D-ZW and S-FZ wrote the manuscript.

## Conflict of Interest Statement

The authors declare that the research was conducted in the absence of any commercial or financial relationships that could be construed as a potential conflict of interest.
